# Supplementary figures and images for: scCaT: An explainable capsulating architecture for sepsis diagnosis transferring from single-cell RNA sequencing
Source: PLoS Comput Biol. 2024 Oct 21;20(10):e1012083. doi: 10.1371/journal.pcbi.1012083 (PMC11527285; doi:10.1371/journal.pcbi.1012083)

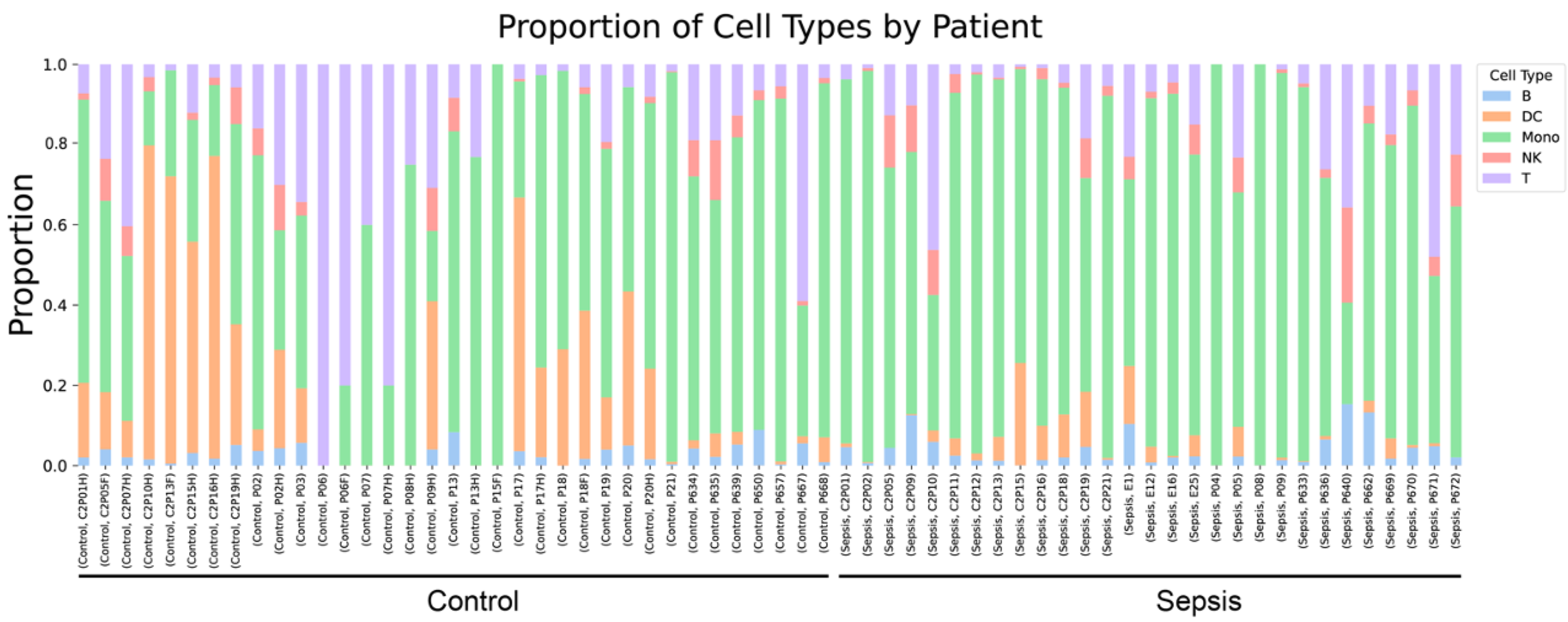

Supplement: S1 Fig — (TIF) [file pcbi.1012083.s004.tif]

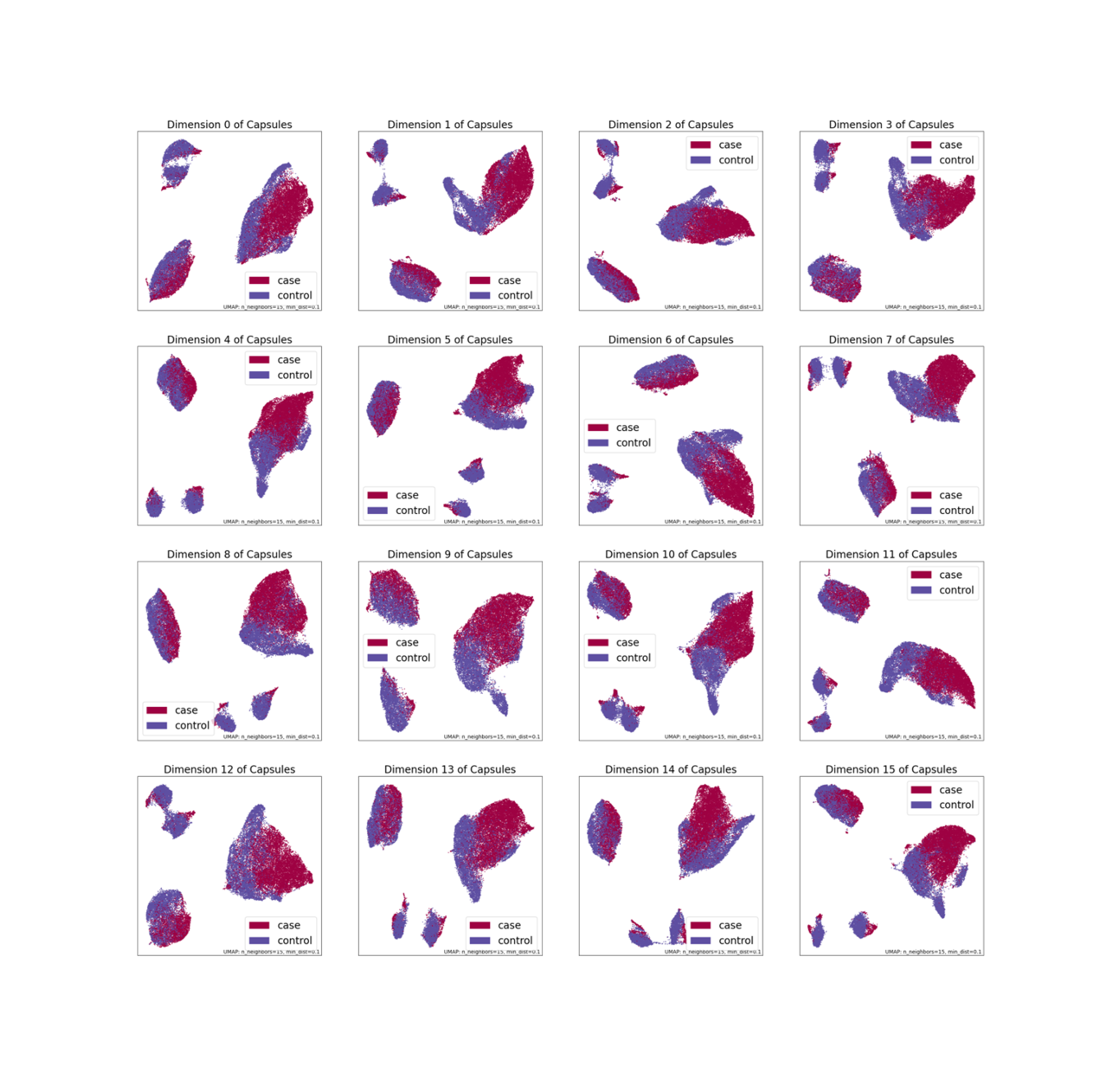

Supplement: S2 Fig — (TIF) [file pcbi.1012083.s005.tif]

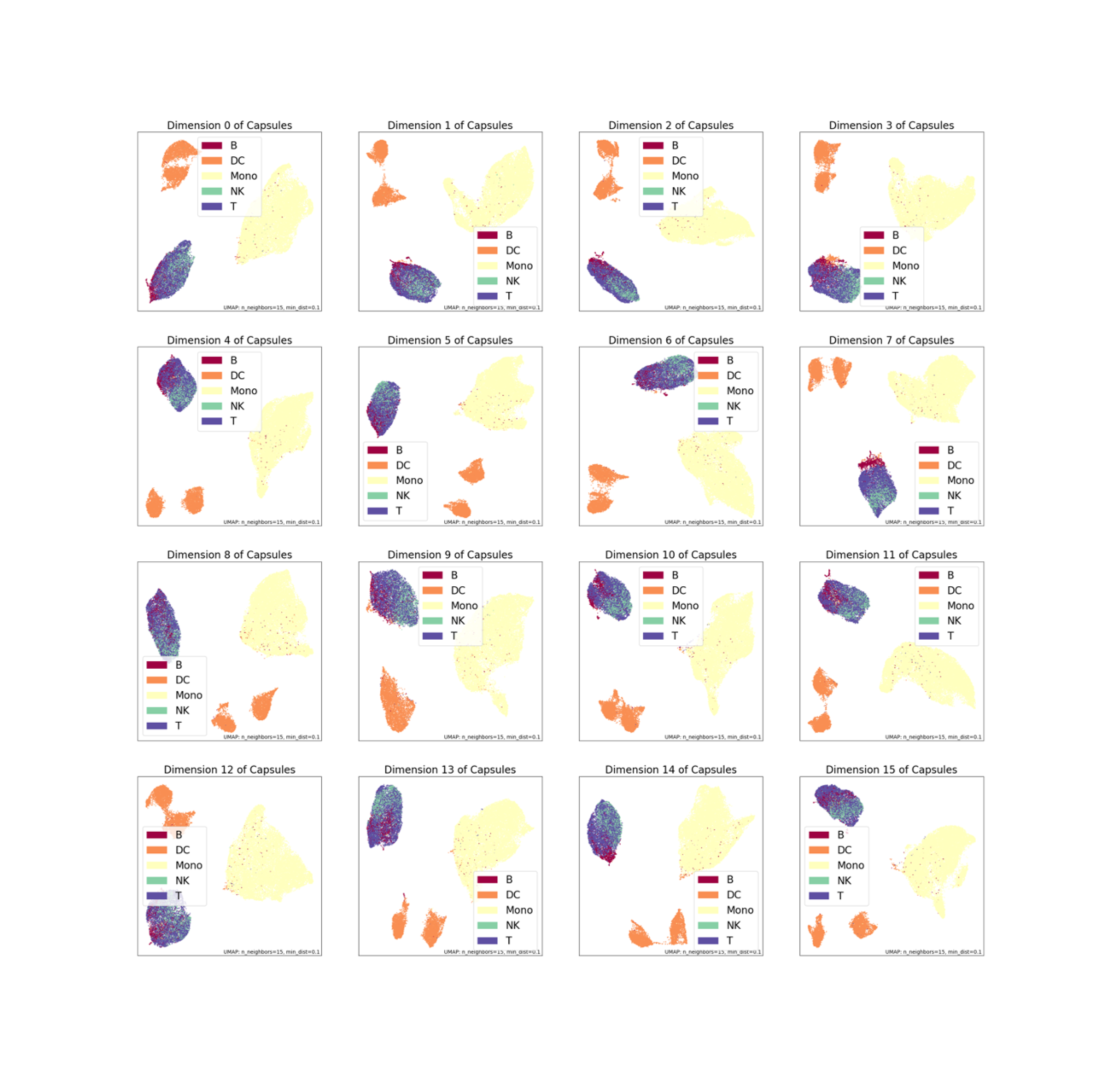

Supplement: S3 Fig — (TIF) [file pcbi.1012083.s006.tif]

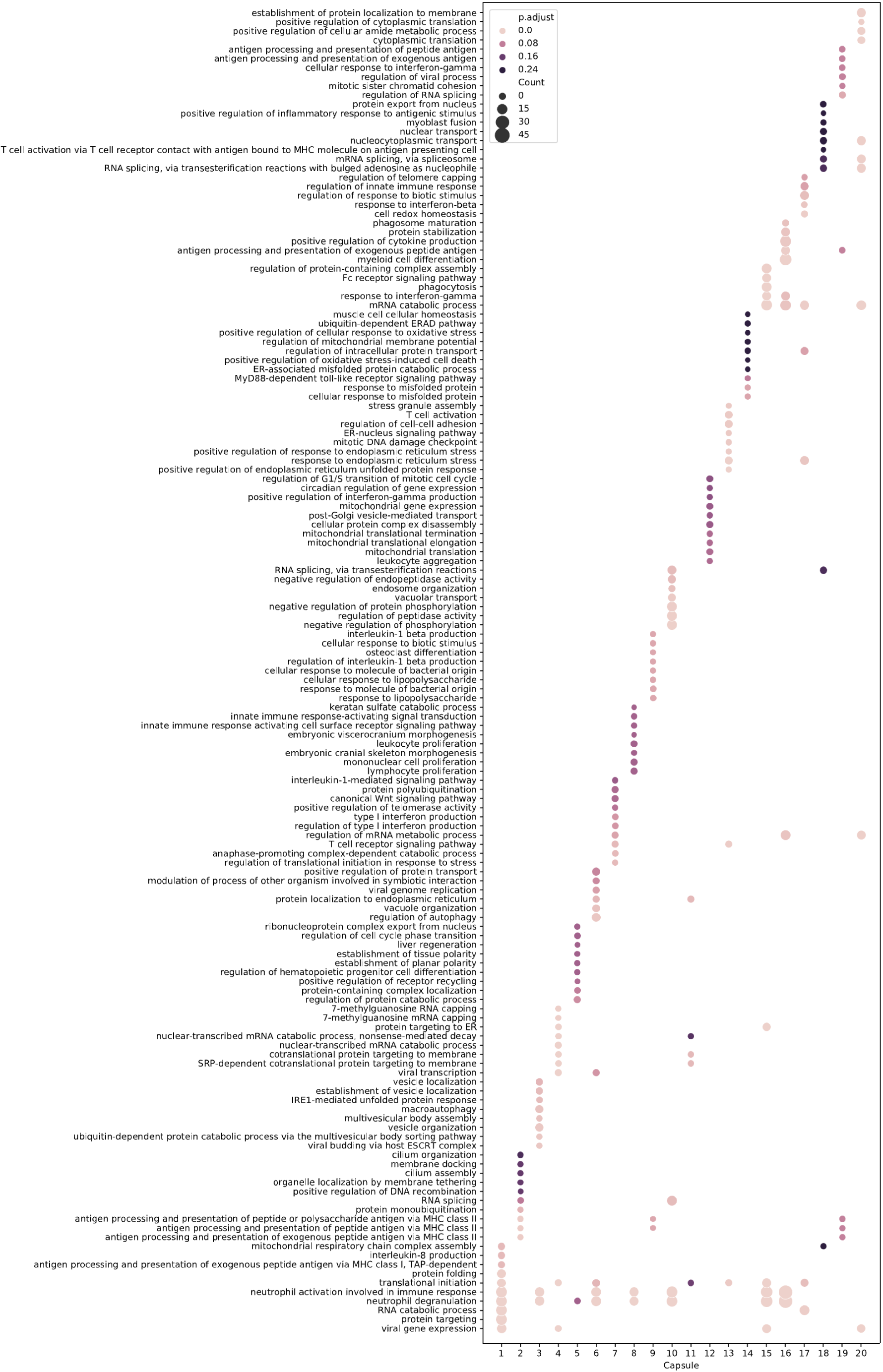

Supplement: S4 Fig — The biological pathways (Y axis) that the twenty capsules (from No.1 to No.20 on X axis) enriched using Gene Ontology (GO). (TIF) [file pcbi.1012083.s007.tif]

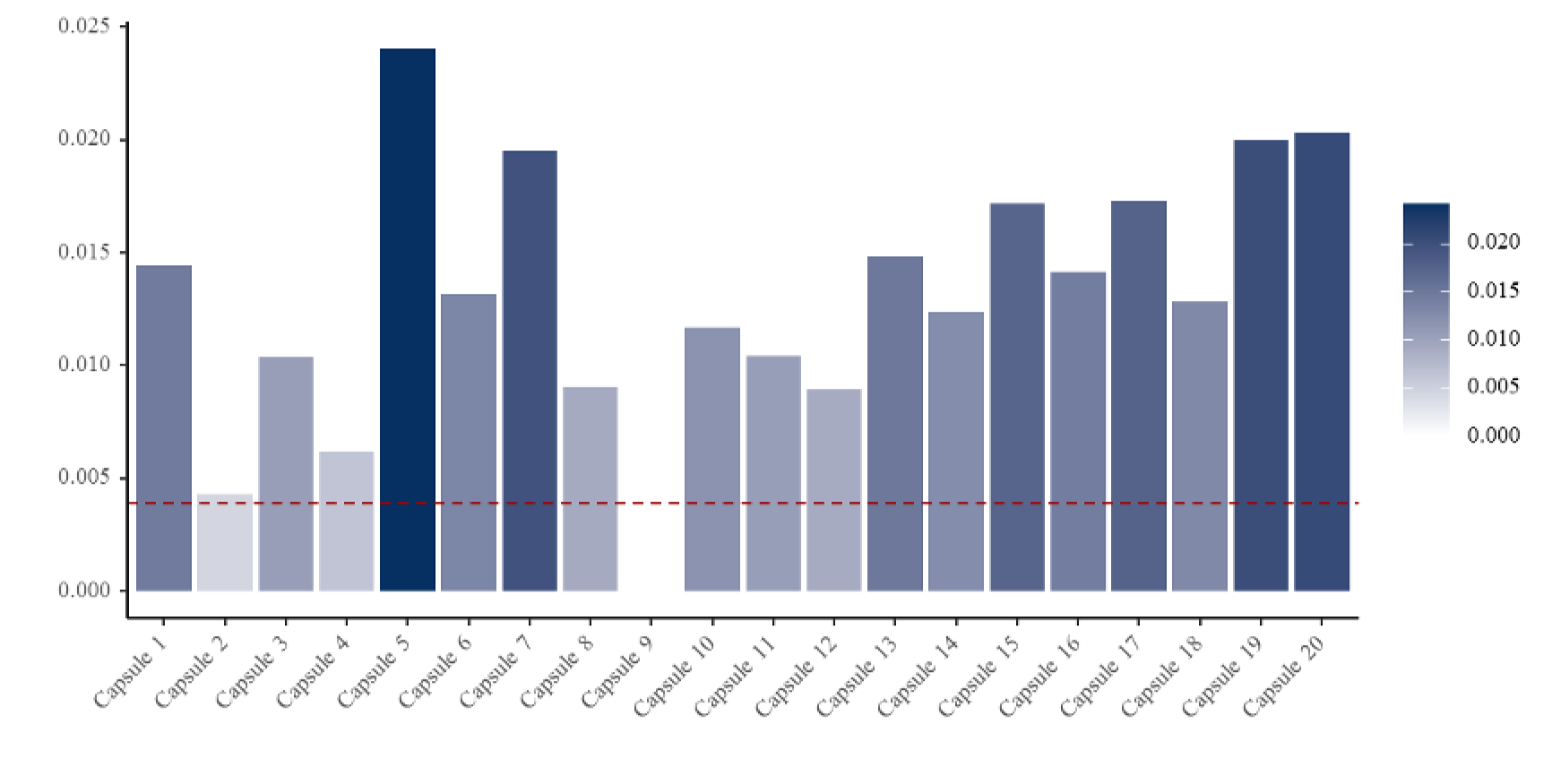

Supplement: S5 Fig — (TIF) [file pcbi.1012083.s008.tif]

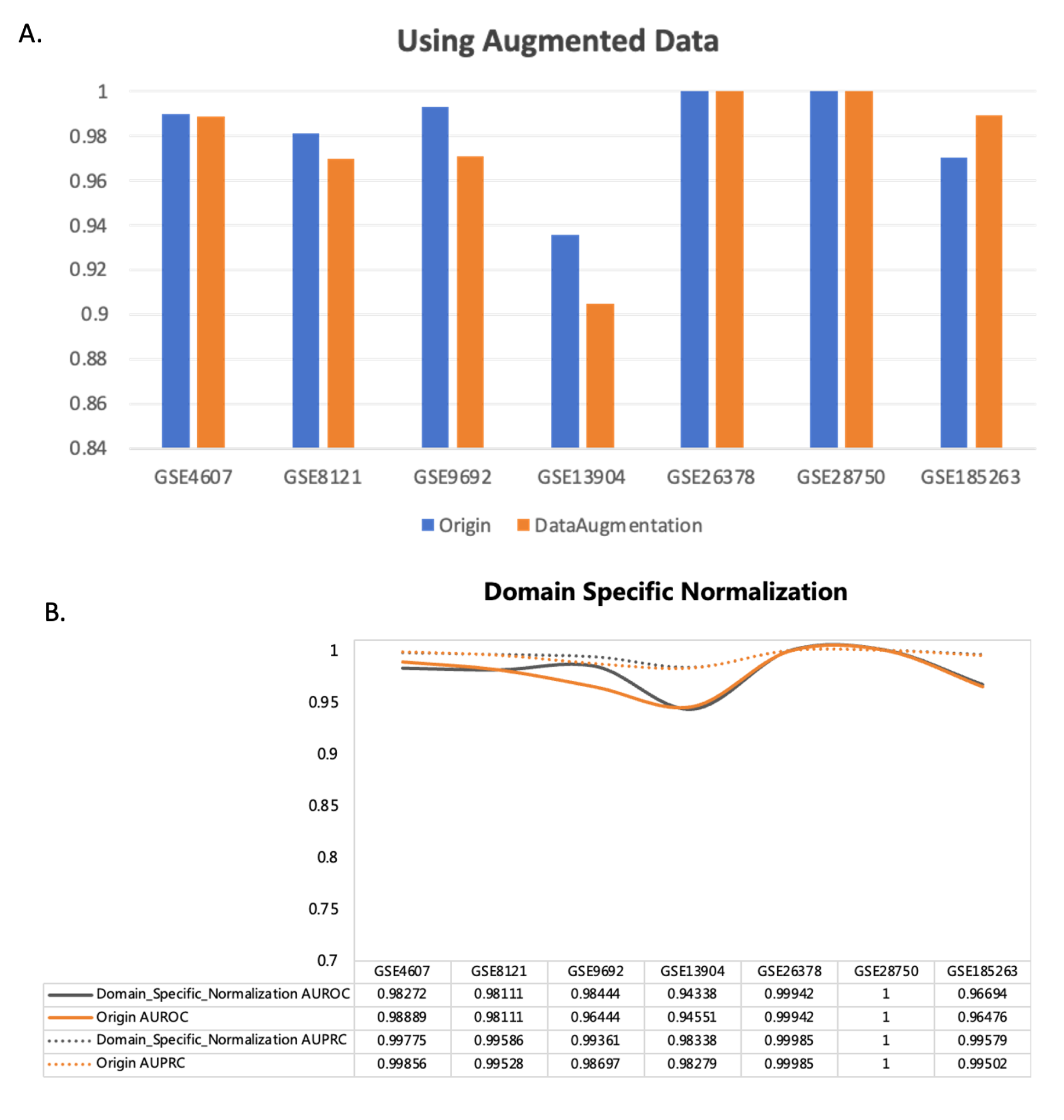

Supplement: S6 Fig — A. The performance of using data augmentation. B. The performance of using domain-specific normalization called adaptive instance normalization. (TIF) [file pcbi.1012083.s009.tif]

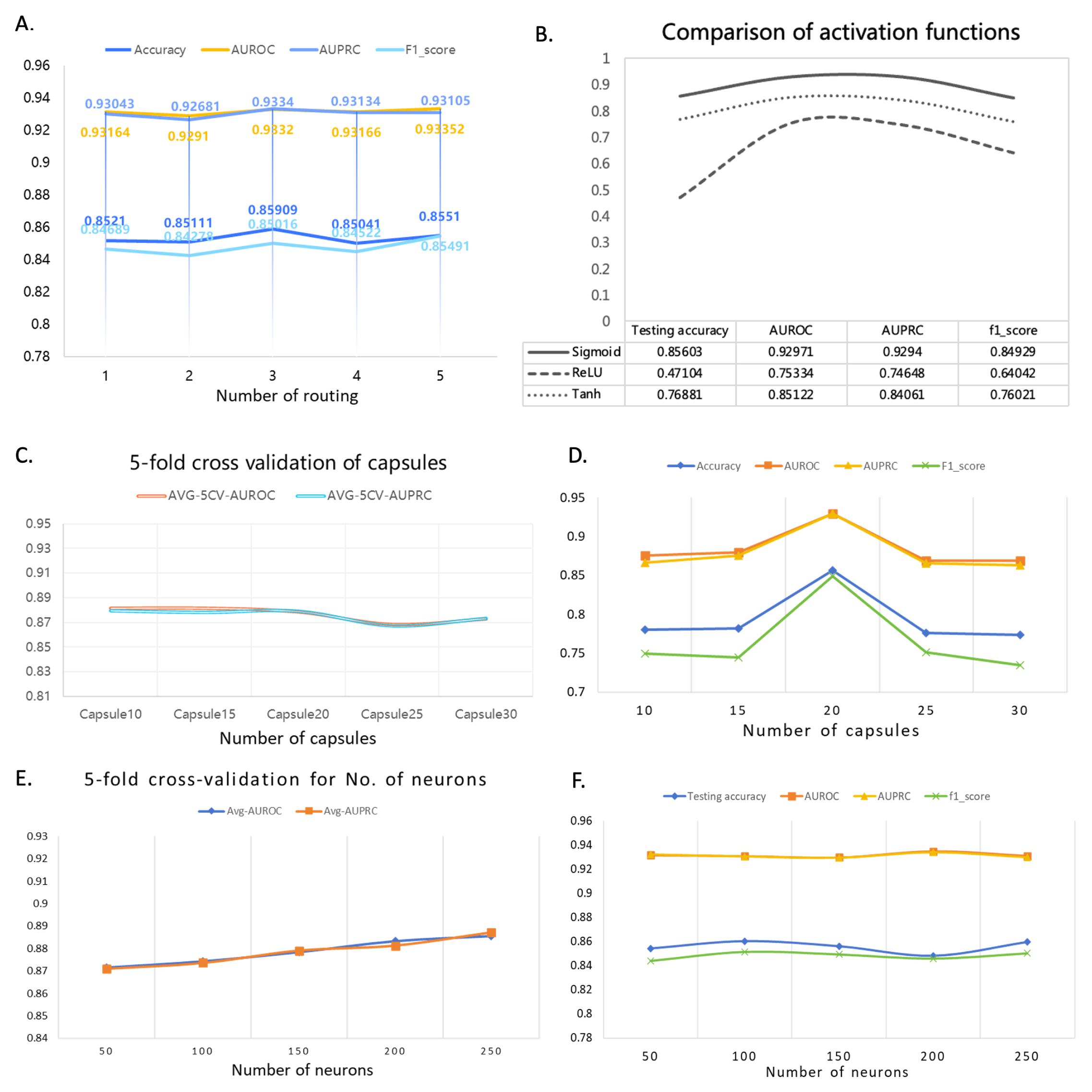

Supplement: S7 Fig — A. The performance of different numbers of routing iterations. B. The performance of different activation functions. C-D. The performance of different capsules. E-F. The performance of different neurons in the dense layer. (TIF) [file pcbi.1012083.s010.tif]
